# Identification of Risk Genes Associated with Myocardial Infarction—Big Data Analysis and Literature Review

**DOI:** 10.3390/ijms232315008

**Published:** 2022-11-30

**Authors:** Cosmin Tirdea, Sorin Hostiuc, Horatiu Moldovan, Alexandru Scafa-Udriste

**Affiliations:** 1 Department of Legal Medicine and Bioethics, Faculty of Stomatology, Carol Davila University of Medicine, 050474 Bucharest, Romania; 2 Department of Cardiac Surgery, Faculty of Medicine, Carol Davila University of Medicine, 050474 Bucharest, Romania; 3 Clinical Emergency Hospital Bucharest, 014461 Bucharest, Romania; 4 Department Cardiology, Faculty of Medicine, Carol Davila University of Medicine, 050474 Bucharest, Romania

**Keywords:** gene, myocardial infarction, risk, association

## Abstract

Acute myocardial infarction occurs when blood supply to a particular coronary artery is cut off, causing ischemia or hypoxia and subsequent heart muscle destruction in the vascularized area. With a mortality rate of 17% per year, myocardial infarction (MI) is still one of the top causes of death globally. Numerous studies have been done to identify the genetic risk factors for myocardial infarction, as a positive family history of heart disease is one of the most potent cardiovascular risk factors. The goal of this review is to compile all the information currently accessible in the literature on the genes associated with AMI. We performed a big data analysis of genes associated with acute myocardial infarction, using the following keywords: “myocardial infarction”, “genes”, “involvement”, “association”, and “risk”. The analysis was done using PubMed, Scopus, and Web of Science. Data from the title, abstract, and keywords were exported as text files and imported into an Excel spreadsheet. Its analysis was carried out using the VOSviewer v. 1.6.18 software. Our analysis found 28 genes which are mostly likely associated with an increased risk for AMI, including: PAI-1, CX37, IL18, and others. Also, a correlation was made between the results obtained in the big data analysis and the results of the review. The most important genes increasing the risk for AMI are lymphotoxin-a gene (LTA), LGALS2, LDLR, and APOA5. A deeper understanding of the underlying functional genomic circuits may present new opportunities for research in the future.

## 1. Introduction

Myocardial infarction (MI) is the interruption of the blood flow to a certain coronary artery that results in necrosis of the heart muscle in the vascularized area [1,2]. MI still represents one of the leading causes of death worldwide [3]. The latest results published by the GBD (Global Burden of Disease) for 2019 showed a 16.17% rate of deaths from cardiac causes globally [4].

Myocardial infarction is difficult to diagnose since it occurs suddenly and unexpectedly. Hypertension, circulating blood lipid levels [5], smoking [6], excessive drinking [7], oral contraceptives [8], high anthocyanin intake [9], HIV infection [10], and a family history or genetic abnormalities are all known cardiovascular risk factors for ischemic heart disease [1]. As a positive family history is one of the most significant risk factors for heart disease, numerous studies have been conducted to characterize the genetic profile of myocardial infarction [1]. For example, Helgadottir et al. [11] found that arachidonate 5-lipoxygenase-activating protein variations play a role in the etiology of myocardial infarction by enhancing arterial wall inflammation and leukotriene generation. Do et al. [12] discovered that multiple rare alleles of the low-density lipoprotein receptor and apolipoprotein A5 increase the risk of myocardial infarction, and a meta-analysis revealed that the rs671 aldehyde dehydrogenase 2 family (mitochondrial) polymorphism increases the risk of myocardial infarction [13]. In a case-control study conducted on a group of over 8000 people from five different ethnic groups, the risk factors for MI were linked to 13 popular SNPs. Anand et al. [14] found that single nucleotide polymorphisms (SNPs) associated with Apo B levels led to an increased risk of MI, even though such a connection has not been found for Apo A1. In the same study, performed on a multiethnic group of subjects, the Apo E isoform and two common low-density lipoprotein receptor variations (rs1433099 and rs6511720) modified the risk of MI [14].

All available data suggests that a greater knowledge of the underlying functional genomic pathways may open new avenues for neutralizing a huge proportion of the population’s hereditary predisposition to MI. This review aims to identify, in an unbiased manner, all the associations between genetic abnormalities and myocardial infarction documented in the literature using big data analysis. As a secondary objective, we aimed to see if there was homogeneity in the findings on the three main medical databases of scientific articles (PubMed, Web of Science, and Scopus).

## 2. Results

**Figure 1:**
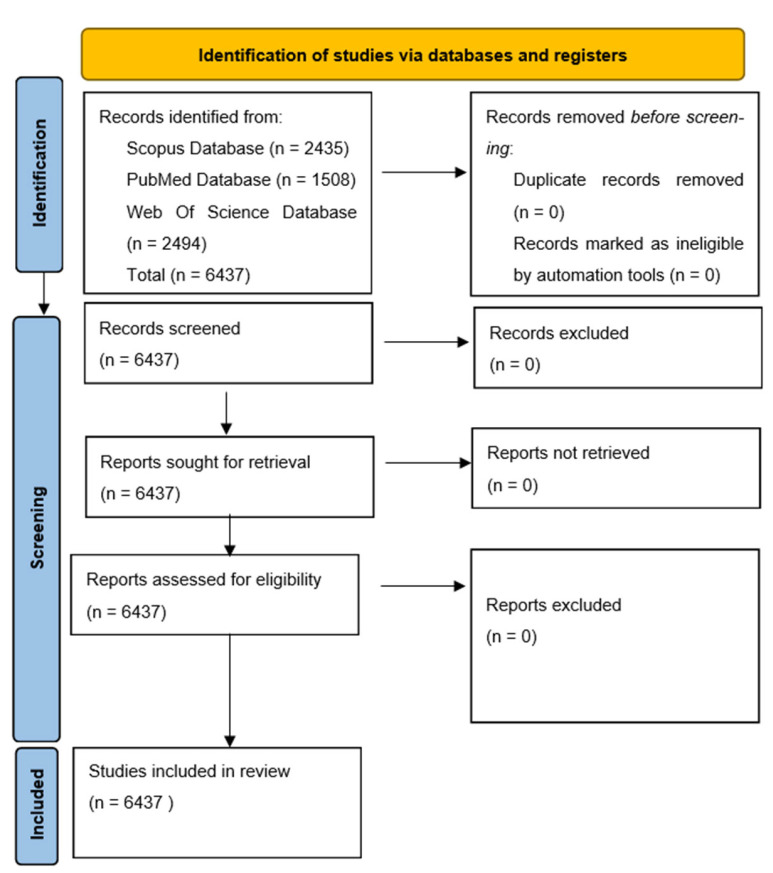
PRISMA Chart [15].

A total of 6436 articles (2435 on Scopus, 1508 on PubMed, and 2494 on Web of Science) that met the search criteria were found on all three search engines, and none were excluded, as can be seen in the PRISMA flow diagram below (Figure 1).

**Figure 2:**
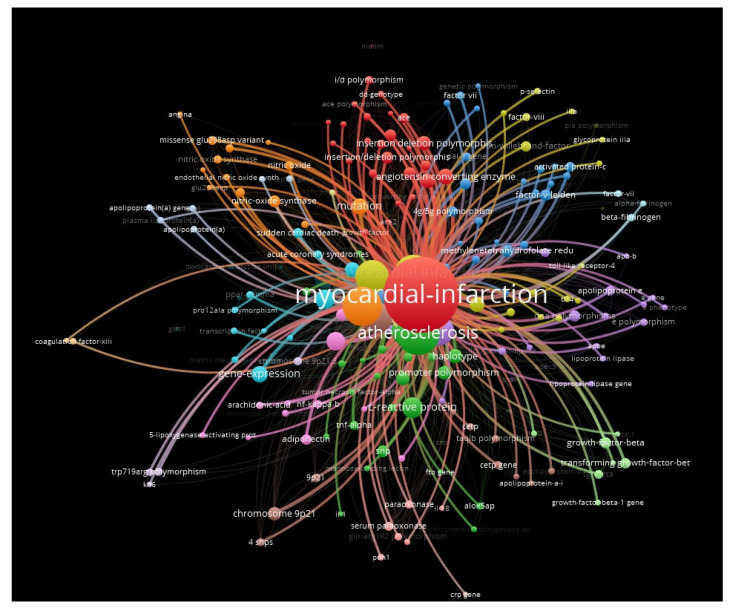
Web of Science map and network.

**Figure A1:**
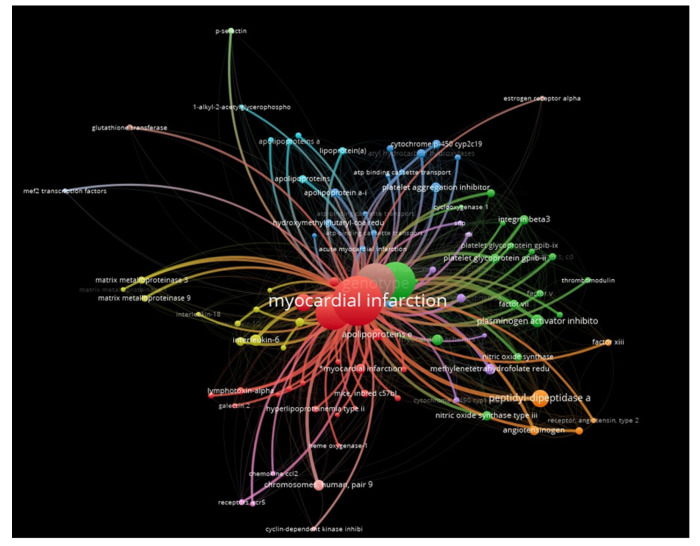
PubMed map and network.

**Figure A2:**
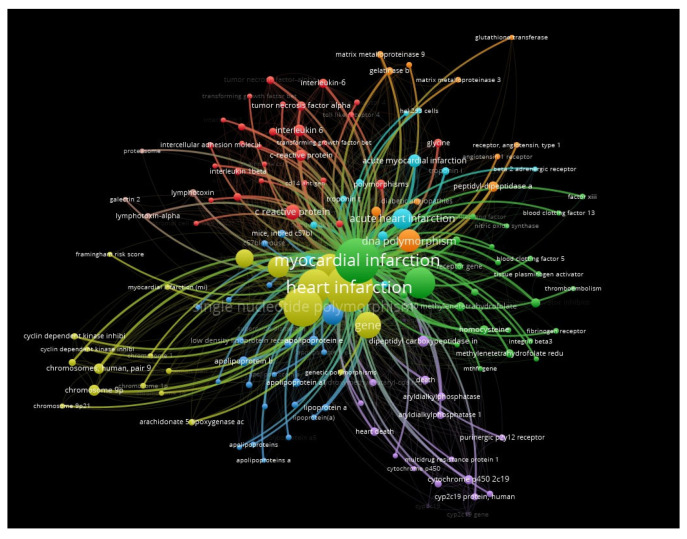
SCOPUS map and network.

The results obtained after the analysis can be seen in the figures below (Figure 2, Figure A1 and Figure A2 from Appendix A).

The program divided the topics of interest found in the database into clusters. Seventeen clusters were obtained, ordered according to the number of connections. “Myocardial infarction” was the largest cluster with 28 gene connections, as can be seen in the figure above (Figure 2) represented by the color red.

According to the big data analysis performed on the database, the direct links with high-risk genes/alleles in the production of a myocardial infarction are represented by: PAI-1, $PI^{A2}$, R353Q, H7H5, H7H6, C242T, Stromelysin-1, IL genes 5A-1171/6A, LTA, LGALS2, AT2 (−1332 G/A), RS671, LDLR, AKAP12, OR8D2, GLRA2, G894T, pro12ala, factor V genes, 4G/5G, 5’-franklin region from nitric-oxide synthase gene, ppar-gamma, glu298asp variant, and PCSK9.

Based on the graphical representation, there were no obvious differences regarding the density of these genes in the studied databases.

## 3. Discussion

The most important genes/alleles identified as being associated with acute myocardial infarction are listed in Table 1 below. A brief description and the possible mechanism of involvement in myocardial infarction were also listed.

Most studies actually analyzing genes associated with an increased risk for MI began to be published in 1995, when Eriksson et al. [16] proved that subjects with coronary heart disease showed increased plasminogen-activator inhibitor 1 (PAI-1) activity, this being an independent risk factor for this disorder. Higher plasma PAI-1 activity is linked to the 4G allele of a recently found frequent 4/5-guanine-tract (4G/5G) polymorphism in the PAI-1 promoter [17]. Patients with myocardial infarction before the age of 45 have a considerably greater prevalence of the 4G allele compared to population-based controls. Both alleles bind to a transcriptional activator, but the 5G allele also binds to an overlapping binding site for a repressor [16]. The baseline level of PAI-1 transcription is raised in the absence of binding repressors [16]. The information presented by Eriksson et al. [16] in 1995 was later confirmed by other studies [18,19] and was clearly identified as an important association in our analysis.

In 1996, Weiss et al. [20] investigated the association between the $PI^{A2}$ polymorphism allele of the platelet glycoprotein IIIa gene and acute coronary syndromes by conducting a case-control study of 71 patients with myocardial infarction or unstable angina and 68 inpatient controls without known heart disease. In the development of acute coronary syndromes, platelets play a significant role. $PI^{A2}$ allele has been linked to immune-mediated platelet damage disorders and thrombosis risk is rising because of $PI^{A2}$ polymorphism. $PI^{A2}$ was found to be 2.1 times more common in case patients than in controls (39.4 percent vs. 19.1 percent, *p* 0.01). $PI^{A2}$ was found in 50% of patients whose sickness began before the age of 60, which was 3.6 times higher than the incidence of $PI^{A2}$ in control participants under 60 years of age (13.9 percent, *p* = 0.002). The $PI^{A2}$ polymorphism of the glycoprotein IIIa gene was found to have a strong link to acute coronary thrombosis, and this link was highest in people who had coronary events before the age of 60 [20]. In another study conducted by Ridker et al. [21] in 1997, it was observed that in a cohort of approximately 15,000 men, the $PI^{A2}$ allele of the glycoprotein IIIa gene was not associated with an increased risk of myocardial infarction [21]. Therefore, the information presented in the two studies is contradictory, but we cannot overlook the fact that the second study includes only men. The information available in the literature regarding this allele is still uncertain, although the big data analysis showed an association between the $PI^{A2}$ allele and myocardial infarction.

In 1998, a case-control study performed by Iacoviello et al. [22] showed that the RR genotype was related to the highest risk of the R353Q polymorphism of the coagulation factor VII gene, followed by the RQ genotype, and finally the QQ genotype *(p* = 0.001). The combined H7H5 and H7H6 polymorphisms for the polymorphism affecting hypervariable region 4, H6H5 genotypes were related to the highest risk, followed by H6H6, H6H7, and H7H7 genotypes in that order (*p* = 0.001) [22]. Also, a study led by Lindman in 2004 [23] supports the conclusions drawn by Iacovellio in 1998 regarding the RQ and RR genotypes. The conclusions reached in the case-control study are also supported by the links from the data analysis.

Oxidative stress in the vasculature has been linked to the pathophysiology of coronary artery disease (CAD). NADH/NADPH oxidase is a key enzyme for superoxide production in the vasculature. An essential part of NADH/NADPH oxidase, p22 phox, exhibits polymorphism in four different ways. The C242T polymorphism causes histidine-72 to convert to tyrosine. Restrictions fragment length polymorphism (RFLP) was utilized by Nobutaka et al. [24] in 1998 to determine whether these variations were related to the risk of coronary artery disease. The C242T polymorphism’s TC+TT genotype was discovered to be significantly more prevalent in control persons (*n* = 201) than in CAD patients (*n* = 201). Between control participants and case patients, the odds ratio of the TC+TT versus CC genotype of the C242T polymorphism was 0.49 (95 percent CI, 0.28 to 0.87) for the TC+TT versus CC genotype (*p* = 0.015) [24]. Atherosclerosis is a multifaceted disease marked by chronic inflammatory alterations in the arterial wall and a lack of normal physical and biochemical connections between endothelial and smooth muscle cells. In order to determine whether there was a chance of genotypic segregation in persons with atherosclerotic plaque, Boerma et al. [25] looked for structural polymorphisms in connexin 37, a gap junctional protein produced exclusively in endothelial cells. At codon 319 (cx37 * 1 cx37 * 2), the C1019-T mutation causes a proline-to-serine switch. A restriction fragment length polymorphism (RFLP) assay involving the insertion of a novel Drd I cleavage site in the proline variant revealed a statistically significant over-representation of the cx37 * 1 allele in association with atherosclerotic plaque-bearing individuals (odds ratio for the homozygote = 2.38, X2 = 7.693, *p* = 0.006), when compared to individuals lacking plaque, irrespective of a history of hypertension [25]. Conclusions supported by our big data analysis.

In 2002, Yamada et al. [26] determined the genotypes of 112 polymorphisms in 71 potential genes using a fluorescence- or colorimetry-based allele-specific DNA-primer–probe assay method in 2819 unrelated Japanese patients with myocardial infarction. Additionally, they found that the genotypes of the stromelysin-1 genes (5A-1171/6A and MMP-3 polymorphisms) may be effective in detecting the hereditary risk of myocardial infarction and, therefore, may aid in primary prevention, in addition to the connexin-37 and plasminogen-activator inhibitor type 1 genes’ implications in MI [26]. In 2004, Lanfear et al., published a paper with similar results regarding stromelysin-1 gene polymorphisms in the African-American population [27]. The information presented by Yamada et al. [26] in 2002 is confirmed by the analysis of the data.

In 2005, Tiret et al. [28] published a study conducted in 1288 individuals with coronary artery disease who were prospectively tracked for a median of 5.9 years and in which twenty-two polymorphisms were genotyped. Cardiovascular fatalities during the first four years of follow-up were predicted by baseline IL-18 levels. After controlling for cardiovascular risk factors, IL-18 levels and cardiovascular mortality were connected by IL-18 haplotypes (*p =* 0.002) (*p =* 0.006). In people with coronary artery disease, variations in the IL18 gene consistently affect circulating levels of IL-18 and clinical outcomes, supporting the idea that IL-18 contributes to atherosclerosis and its consequences [28]. Also, a meta-analysis of 15 studies published by Lian et al., suggested that the -137 polymorphism and the -607 polymorphism in the IL-18 promoter were associated with CAD and its consequences. [29] The information presented by Tiret et al. [28] in 2005 is confirmed by the analysis of the data.

Four gene variants were found to be associated with MI by Shiftman et al. [30] (*p* = 0.05; false-discovery rate = 10%) and shared the same risk allele as another two studies conducted by them. The cytoskeletal protein palladin (KIAA0992 [OR 1.40]), a tyrosine kinase (ROS1 [OR 1.75]), and two G protein–coupled receptors (TAS2R50 [OR 1.58] and OR13G1 [OR 1.40]) are all encoded by these gene variants; all Ors are for carriers of two versus zero risk alleles [25].

Ozaki et al. [31] used 92,788 gene-based single-nucleotide polymorphism (SNP) markers in a large-scale case-control association analysis to elucidate the genetic origins of myocardial infarction. On chromosome 6p21, they found functional SNPs in the lymphotoxin-a gene (LTA) that contributed to myocardial infarction susceptibility. The galectin-2 protein was also identified as a ligand for the LTA protein. A functional SNP in LGALS2 encoding galectin-2, which resulted in altered LTA secretion, was likewise linked to an increased risk of myocardial infarction, according to the study [31]. Ozaki et al.’s [31] case-control analysis led to conclusions sustained by our big data analysis.

In 2007, Helgadottir et al. [32] studied the association between myocardial infarction (MI) and a common sequence variant on chromosome 9p21. The newly identified variant, which was found close to the tumor suppressor genes CDKN2A and CDKN2B, was substantially associated with the disease. About 21% of the population is homozygous for this mutation, and their risk of myocardial infarction is 1.64 times higher than that of noncarriers. The risk was 2.02 times higher in early-onset cases than in late-onset cases. For MI in general, the population attributable risk is 21%, and for early-onset instances, it is 31% [32]. Yuan et al. [33] support the conclusions about the CDKN2B gene.

Alfakih et al. [34] also looked into 885 families in 2007 in which at least one sibling had premature CAD and at least one sibling was unaffected. The patients’ genotypes were determined by restriction enzyme digestion of an initial 310-bp PCR fragment that contained the AT2 (-1332 G/A) gene. They found solid proof that the AT2 (-1332 G) gene and early CAD are related. A highly significant outcome in men was the catalyst for this. They also found evidence of a statistically significant link between the X-linked AT2 (-1332 G/A) polymorphism and early CAD, as well as evidence of a statistically significant link with stenotic atherosclerosis [34]. The information presented by Alfakih et al. [34] in 2007 is confirmed by the analysis of the data.

A meta-analysis on the relationship between genetic variants in ADH and ALDH2 and the risk of coronary artery disease and myocardial infarction was carried out by Han et al. [13] in 2013. The meta-analysis found a significant association between the mutant genotypes (GA+AA) of the rs671 polymorphism in the ALDH2 gene and an increased risk of CAD and MI (RR = 1.20, 95 percent CI: 1.03–1.40, *p* = 0.021; MI: RR = 1.32, 95 percent CI: 1.11–1.57, *p* = 0.002). The incidence of CAD or MI was not significantly correlated with genetic variations in ADH, though. In conclusion, the ALDH2 rs671 polymorphism may be linked to an increased risk of CAD and MI [13]. The results obtained in the meta-analysis are also supported by the links in the data analysis.

Do et al. [12] sequenced the protein-coding portions of 9793 genomes from MI patients and MI-free controls at an early age (<50 years for men and <60 years for females). Carriers of the low-density lipoprotein receptor (LDLR) had a 2.4-fold increased risk of MI compared to controls (3.1 percent of cases versus 1.3 percent of controls), while carriers of the LDLR null allele had an even higher risk (a 13-fold difference). MI risk was increased by 2.2-fold in people who carried rare nonsynonymous mutations in apolipoprotein A-V (APOA5 promoter region) (1.4 percent of cases versus 0.6 percent of controls). These findings imply that, in addition to LDL cholesterol, abnormal triglyceride-rich lipoprotein metabolism has a role in MI risk [12]. Conclusions supported by our big data analysis.

In 2017, Yang et al. [1] conducted a study and identified two risk genes: A-kinase anchoring protein 12 (AKAP12) and glycine receptor 2 (GLRA2). As a result, AKAP12 and GLRA2 may have a role in the progression of myocardial infarction by affecting cardiac contractility [1]. The information presented by Yang et al. [1] is confirmed by our analysis of the data.

Our analysis is based on a graphical representation of the associations between MI and various genetic profiles. A more in-depth, quantitative approach should be performed to evaluate the actual risk increase associated with distinct genetic profiles. Any analysis of this type (including this one) is affected by a non-recency bias: associations studied earlier usually generate more subsequent research to confirm the findings than associations studied more recently. This would lead to a potential underrepresentation of recently identified associations. This shortcoming may be solved by either performing studies using a shorter time-frame, or by performing in-depth, narrative, or systematic reviews of recent literature in the field.

Considering that studies showed a multitude of genes that can be associated with myocardial infarction, genetic test kits available on the market were designed. The kits evaluate over 6000 genes, including genes that were presented in this literature review. Among the tested genes we list: APOE, LRP6, LDLR, APOB, PCSK9, ACTC1, CALR3, CAV3, CSRP3, GLA, JPH2, LAMP2, LIPA, MYBPC3, MYH6, MYH7, MYL2, MYL3, MYLK2, MYOZ2, NEXN, PLN, PRKAG2, SLC25A4, TNNC1, TNNI3, TNNT2, TPM1, TTN, TTR, and VCL.

## 4. Materials and Methods

We searched PubMed’s, Web of Science, and Scopus databases for articles that met the study’s objectives. The following keywords were used for the generic search: “myocardial infarction”, “genes”, “involvement”, “association”, “risk”, “genes involved in myocardial infarction”, and “genes associated with increased risk of myocardial infraction”. After performing the search, the option chosen was to display only the articles that have the abstract available for viewing. Another inclusion criterion was the display of studies published starting with the year 1995. The next step was to save all the results offered by the search engine in a CSV document. This algorithm for searching and displaying the results was applied to all the databases mentioned above.

The analysis was carried out using VOSviewer version 1.6.18 from Leiden University’s Centre for Science and Technology Studies (CWTS), which establishes maps and networks based on the association of terms in relation to the user’s subject of interest. In the network visualization, each item is represented by a label and a circle, whose diameter is proportionate to the number of mentions of that keyword in the mined texts.

## 5. Conclusions

The big data analysis revealed the most relevant genes/alleles that present an increased risk of myocardial infarction, represented by: PAI-1, Stromelysin-1, 5A-1171/6A, LTA, LGALS2, AT2 (−1332 G/A), RS671, LDLR, AKAP12, OR8D2, GLRA2, the 5’-franklin region of the nitric-oxide synthase gene. Looking back, research carried out from 1995 until now has confirmed some of the genes discovered by us through the analysis. This suggests that conducting new research on population subgroups can enrich our knowledge about the mechanisms of myocardial infarction.

## Figures and Tables

**Table 1:** The most important genes/alleles associated with acute myocardial infarction.

Genes/Alleles	Description	Possible Implication in MI
PAI-1	Encodes a member of the serine proteinase inhibitor (serpin) superfamily	Reduced fibrinolytic capacity
$PI^{A2}$	Membrane receptor for fibrinogen and von Willebrand factor (platelet glycoprotein IIIa gene)	Platelet aggregation at the site of a ruptured coronary atherosclerotic plaque
Alleles: R353Q H7H5, H7H6	Coagulation Factor VII regulator gene	Factor VII clotting activity
Stromelysin-1	Cleaves a number of extracellular matrix and non-extracellular matrix proteins	Progression of coronary atherosclerosis
IL Genes	Proinflammatory cytokine involved in both innate and acquired immune responses	Atherosclerosis
LTA LGALS2	Lymphotoxin-alpha (LT-α) or tumor necrosis factor-beta (TNF-β)	
AT2 (-1332 G/A)	Angiotensin II type 2 (AT2) receptor	Stenotic atherosclerosis
RS671		Coronary artery disease
LDLR	Low-density lipoprotein receptor	Atherosclerosis
AKAP12	Scaffolding proteins that regulate the cellular cyclic AMP response	Influencing cardiac contractility
OR8D2	G-protein-coupled receptors	Atherosclerosis
GLRA2	Glycine receptor	Influencing cardiac contractility

## Data Availability

Not applicable.
